# Assessment of orthologous splicing isoforms in human and mouse orthologous genes

**DOI:** 10.1186/1471-2164-11-534

**Published:** 2010-10-01

**Authors:** Federico Zambelli, Giulio Pavesi, Carmela Gissi, David S Horner, Graziano Pesole

**Affiliations:** 1 Dipartimento di Scienze Biomolecolari e Biotecnologie, Università degli Studi di Milano, Via Celoria 26, 20133 Milano, Italia; 2 Istituto Biomembrane e Bioenergetica, Consiglio Nazionale delle Ricerche, via Amendola 165/A, 70125 Bari, Italia; 3 Dipartimento di Biochimica e Biologia Molecolare "E. Quagliariello", Università degli Studi di Bari, Via Orabona 4, 70126 Bari, Italia

## Abstract

**Background:**

Recent discoveries have highlighted the fact that alternative splicing and alternative transcripts are the rule, rather than the exception, in metazoan genes. Since multiple transcript and protein variants expressed by the same gene are, by definition, structurally distinct and need not to be functionally equivalent, the concept of gene orthology should be extended to the transcript level in order to describe evolutionary relationships between structurally similar transcript variants. In other words, the identification of true orthology relationships between gene products now should progress beyond primary sequence and "splicing orthology", consisting in ancestrally shared exon-intron structures, is required to define orthologous isoforms at transcript level.

**Results:**

As a starting step in this direction, in this work we performed a large scale human- mouse gene comparison with a twofold goal: first, to assess if and to which extent traditional gene annotations such as RefSeq capture genuine splicing orthology; second, to provide a more detailed annotation and quantification of true human-mouse orthologous transcripts defined as transcripts of orthologous genes exhibiting the same splicing patterns.

**Conclusions:**

We observed an identical exon/intron structure for 32% of human and mouse orthologous genes. This figure increases to 87% using less stringent criteria for gene structure similarity, thus implying that for about 13% of the human RefSeq annotated genes (and about 25% of the corresponding transcripts) we could not identify any mouse transcript showing sufficient similarity to be confidently assigned as a splicing ortholog. Our data suggest that current gene and transcript data may still be rather incomplete - with several splicing variants still unknown. The observation that alternative splicing produces large numbers of alternative transcripts and proteins, some of them conserved across species and others truly species-specific, suggests that, still maintaining the conventional definition of gene orthology, a new concept of "splicing orthology" can be defined at transcript level.

## Background

Alternative splicing (AS) is emerging as a key mechanism for the expansion of transcriptome and proteome complexity in eukaryotes [1]. Extensive investigations carried out so far, using sequence and microarray data, have shown different levels of AS in eukaryotes [2,3] with a considerable fraction of AS splicing events resulting species- or lineage-specific at level of comparisons involving even very closely related species such as human and chimp [4], but also human and mouse [5-7], other mammals [8,9], fruit flies [10] or plants [11,12]. It is now well known that alternative splicing (AS) affects over 90% of genes in humans and in other organisms [13-15], and accounts for the increase of at least one order of magnitude in transcriptomic and proteomic complexity. A typical human gene, then, produces multiple transcript isoforms (ten per gene, on average), that can differ both in their coding and untranslated regions. Thus, protein products with different properties, or transcripts subject to different post-transcriptional regulatory pathways can be generated in different proportions in different tissues, developmental stages or physiological conditions. This widespread prevalence of AS challenges our perception of what might constitute a gene [16-18], and even if does not affect the concept of orthology relationship at the gene level, at the transcript level requires the introduction of a new concept of "splicing orthology" to identify structurally similar splicing variants whose products may also be more likely functionally related.

Functional and evolutionary analyses of genes are usually performed on one or few representatives of their expression products, i.e. transcripts and proteins. Strict orthology of genes requires vertical descent from a common ancestor in the absence of intervening gene duplications, and it is often interpreted as indicating functional equivalence. In practice, two genes α and β are usually annotated as orthologous if their products are the "best reciprocal hits": that is, one of the proteins encoded by the α gene of species A has, as its most similar counterpart in species B, one of the proteins encoded by the β gene, and vice versa.

However, it is known that different gene isoforms may play different, and even antagonistic, functional roles that can also be species-specific [19]. For example, the human caspase 9 gene encodes two alternative products with antagonistic functions: the longer isoform is pro-apoptotic whereas the shorter one, which lacks a functional protease domain due to skipping of four contiguous exons, is anti-apoptotic [20]. While mechanisms of evolution of patterns of alternative splicing are not yet well understood, splicing patterns shared between orthologous genes in closely related organisms are likely to reflect descent from an ancestral splicing pattern. Taken together, these considerations suggest that it is important to extend the notion of orthology from genes to single transcripts, in order to relate structurally similar splicing variants, i.e. those variants sharing the same exon-intron structure and using the same homologous splicing sites. Indeed, the multiple isoforms expressed by two orthologous genes, depending on their sequence/structure similarity in coding and non-coding portions, can be more or less functionally related with some isoform pairs largely functionally equivalent and others possibly species-specific.

Within vertebrates and other major eukaryotic taxa, a remarkable conservation of the exon-intron structure of genes can be observed [21], and this characteristic has been identified as a reliable marker of orthologous genes, even at large evolutionary distances [22]. Considering pairs of orthologous genes, each encoding a set of alternative transcripts, we can detect pairs of transcript variants employing identical exon-intron structures, i.e. orthologous splicing variants, with other variants being species-specific or lacking characterized orthologous counterparts. It is more likely that orthologous splicing variants encode functionally equivalent products, while transcripts with a different exon-intron structure (e.g. differing in the number and/or length of exons) may encode products with different functional features and/or subject to different post-transcriptional regulation.

**Figure 1:**
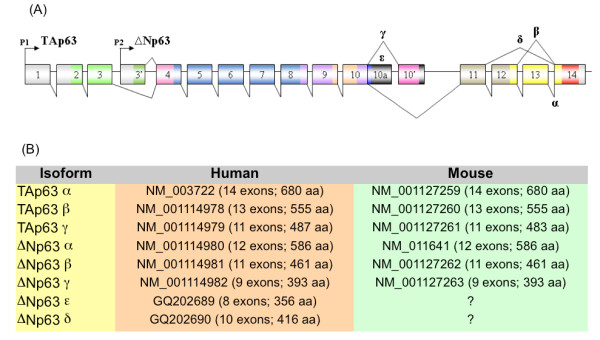
**P63 isoforms in human and mouse**. (A) The structure of human and mouse p63 genes showing the two alternative promoters (P1 and P2) and the alternative splicing events giving rise to the α, β, γ, δ and ε isoforms of the TA and ΔN class. (B) Pairs of orthologous splicing variants in human and mouse.

Human and mouse p63 genes, whose products play key roles in development and differentiation, constitute a typical example. These genes encode multiple isoforms, with distinct function and expression patterns, as a result of the usage of two alternative promoters and alternative splicing [23]. The upstream and downstream promoters encode the TA and ΔN classes of isoforms, each of which in turn includes at least three variants denoted α, β and γ through alternative splicing. Therefore, for this gene we may expect to detect at least six pairs of orthologous splicing variants (Fig. 1). Notably, two additional functional isoforms (δ and ε) have been detected in human, which still do not have known counterparts in mouse [24]. However, while p63 is a well studied example, only a limited number of alternative transcripts are usually reported in general databases such as the NCBI RefSeq collection [25] with the consequence that for orthologous genes different splicing isoforms may be annotated in different organisms.

To investigate the extent of this issue, we used the Exalign algorithm [26] to compare the full set of human gene structures, defined by mapping human RefSeq transcripts to the genome, to their murine equivalents, with a twofold goal: first, to assess if and to which extent current gene annotations are able to incorporate truly orthologous splicing variants; second, to provide a more detailed annotation of actual human-mouse orthologous transcripts, defined as transcripts of orthologous genes exhibiting the same splicing patterns.

## Results

We retrieved a total of 16641 human/mouse orthologous gene pairs from the annotations available in [27], discarding genes without an annotated ortholog in either species. We excluded fully non-coding exons and the non-coding portion of external exons from the comparison, as exons corresponding to 5' and 3' untranslated regions of mRNAs are much more variable in length and often in number, as well as being less consistently annotated. Only RefSeq genes having an annotated transcript with at least four exons and at least two internal fully coding exon in both human and mouse were analysed. This left 12267 orthologous gene pairs. Whenever the restriction to internal coding exons resulted in the presence of redundant transcripts, sharing internal coding exons of the same size and reading frame, we used only one representative in our analyses.

As well as comparing human and mouse RefSeq collections, we also compared the RefSeq transcript collection to the transcripts included in ASPicDB [13], which collects all putative alternative isoforms for a given gene determined by the ASPIC algorithm [28,29]. ASPIC first performs a multiple alignment of all full-length transcripts and ESTs of the corresponding Unigene cluster against the relevant genomic region, and then reconstructs the putative full-length isoforms by combinatorial assembly. ASPicDB contains, for human and mouse genes respectively, over 200,000 and 60,000 mostly novel inferred transcript variants.

For each gene pair, we checked whether each annotated RefSeq transcript showed sufficient structural similarity to a transcript derived from the orthologous gene in the other species for the transcript pair to be considered as orthologous splicing counterparts. To this end, we compared the intron-exon transcript structure of the homologous coding regions with three different criteria, in decreasing order of stringency:

1. The two coding regions are made of the same number of coding exons, and these exons (or the coding part of partially coding exons) have the same size and are preceded by introns in the same phase.

2. The two transcripts have the same number of coding exons and corresponding coding exons (or the coding part of partially coding exons) are: (a) preceded by introns in the same phase, and (b) their length differ by 3, 6, 9, 12 or 15 base pairs.

3. The two transcripts have the same number of coding exons, and corresponding exons (or the coding part of partially coding exons) preserve the phase of the preceding intron, allowing length differences of any size.

**Figure 2:**
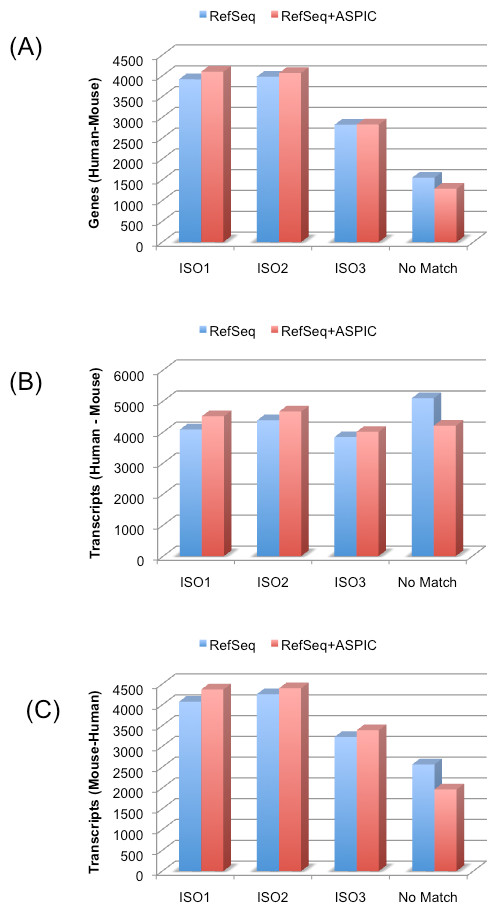
**Splicing orthologs in human and mouse**. (A) Number of human/mouse orthologous gene pairs with transcript pairs fulfilling the three different criteria of splicing orthology (ISO1, ISO2 and ISO3) or giving no match considering only RefSeq or ResSeq+ASPicDB transcripts. (B) Number of human RefSeq transcripts with an orthologous splicing counterpart in mouse RefSeq (or RefSeq + ASPicDB) transcripts, and (C) vice versa for mouse. Gene structure comparisons have been carried out by Exalign software [26].

The overall results of these comparisons are summarized in Figure 2. Moreover, a table indicating for each RefSeq transcript the transcript considered its best structural match from the corresponding orthologous gene is provided in Additional file 1.

As shown in Figure 2A, a total of 1551 orthologous gene pairs (13%) did not produce any pair of RefSeq transcripts satisfying at least one of the criteria defined above: thus all these orthologous gene pairs have different splicing isoforms annotated in RefSeq. Using the most stringent similarity criterion 1 we obtained 3915 genes producing at least one transcript with an identical intron/exon counterpart in the other species (32%). When we applied the less stringent criterion 2 to the remaining genes we identified that a further 32% (3979) of the genes had a transcript with a matching counterpart. Likewise, 2822 gene pairs that did not have any orthologous splicing match according to the two previous criteria produced at least one transcript pair satisfying condition 3 (23%).

When ASPicDB transcripts were included in the comparisons, the number of human genes with an orthologous splicing transcript in mouse rose to 4098, 4067, and 2838 for each of the three criteria, while 1283 (10%) of the human genes still remained without any RefSeq transcript with an orthologous splicing variant of any type in mouse. Likewise, the same numbers for mouse rose to 4115, 4068, and 2896 while 1216 genes (again, about 10%) still lack any orthologous splicing counterpart in human even including the ASPicDB transcripts in the comparison.

At the single transcript level (Figure 2B), we analyzed 17383 non-redundant human RefSeq transcripts: 4511 (25%) had a matching transcript in mouse according to criterion 1, of which 432 were ASPicDB predictions; 4661 (28%) a match of type 2, 283 of which ASPicDB predictions; 4006 (23%) a match of type 3 (169 ASPicDB predictions). Remarkably, 5089 RefSeq human transcripts (29% of the total) did not have any RefSeq counterpart in mouse satisfying any of the three criteria; by including ASPicDB transcripts this number was reduced to 4205, still about 24% of the total (red slice in Figure 2B). That is to say that we could not find any transcript in mouse that could be considered an "orthologous splicing isoform" for about one human RefSeq transcript out of four.

Conversely, in mouse (Figure 2C), of 14120 available non redundant RefSeq transcripts, 4368 (31%) had a matching human transcript of type 1, of which 294 derived from ASPicDB predictions; 4397 (31%) had a match of type 2, 143 of which derived from ASPicDB predictions; 3389 (24%) showed had as best match a counterpart of type 3 (163 ASPicDB predictions). This left 2566 mouse RefSeq transcripts (18% of the total) without any RefSeq counterpart in human, reduced to 1966 (14%, red slice in Figure 2C) if ASPicDB predictions were included.

Thus, for mouse, the resulting percentage of transcripts without an orthologous splicing match is lower than for human (compare the "no match" bars in Figure 2B and 2C), mainly due to the fact that a larger number of human transcripts (RefSeq or ASPicDB prediction) was available for the comparison (17383 against 14120). Notwithstanding the fact that the prevalence of species-specific isoforms between human and mouse remains a controversial subject [5,9,19], analyses incorporating all available EST data indicate that conservation of AS between human and mouse is rather extensive and additional transcript sequence data can be expected to increase the number of identifiable orthologous transcript pairs. Moreover, RefSeq transcript collections (and gene annotations), usually employed to establish and assess orthology relationships, appear to be highly incomplete with respect to alternative splicing. On the other hand, we can still expect quite a sizable number of human or mouse transcripts to remain "species-specific" without a truly "splicing-orthologous" counterpart in the other species.

**Figure 3:**
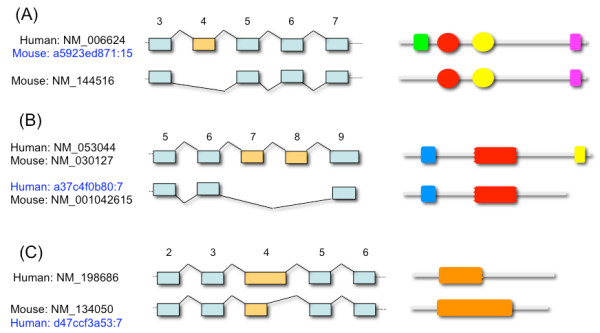
**Examples of structural differences between annotated RefSeq orthologs in human and mouse**. (A) Exalign structural alignment between ZMYND11 RefSeq transcripts NM_006624 (human) and NM_144516 (mouse); the four domains of the protein encoded by NM_006624, as identified by PFAM: from left to right a PHD domain, a bromodomain, a PWWP domain and a MYND finger domain; the three domains of the protein encoded by NM_144516 as identified by PFAM: a bromodomain, a PWWP domain and a MYND finger domain. The mouse isoform annotated in ASPicDB under the signature ID: a5923ed871:15 [39] has a structure identical to human NM_006624. (B) Exalign alignment between HTRA3 RefSeq transcripts NM_053044 (human) and NM_001042615 (mouse); the three PFAM domains annotated on the protein encoded by NM_053044: a Kazal-2 domain, a trypsin domain and a PDZ domain; the two domains of the protein encoded by NM_001042615 identified by PFAM: a Kazal-2 domain and a trypsin domain. The human ASPicDB isoform (signature ID: a37c4f0b80:7) has a structure identical to mouse NM_001042615. (C) Exalign alignment between RAB15 RefSeq transcripts NM_134050 (mouse) and NM_198686 (human); the protein encoded by NM_198686 presents a truncated RAS domain while the protein encoded by NM_134050 shows a complete RAS domain. The human ASPicDB isoform d47ccf3a53:7 has a structure identical to mouse NM_134050. Orthologous isoforms not included in the RefSeq database but supported by transcript evidences and collected in ASPicDB [13] as well as in other alternative splicing databases [38] are indicated in blue by their unique signature ID [39].

Indeed, as shown in the examples of Figure 3 we found many cases where annotated isoforms from orthologous genes showed notably different structures, but where available evidences indicates that iso-orthologous forms are produced *in-vivo*. Figure 3A shows ZMYND11, a gene for which there are two annotated RefSeq transcripts in human (NM_006624 and NM_212479) and only one in mouse (NM_144516). In terms of structure, NM_144516 is more similar to NM_006624 (these two transcript structures are identical, apart from the absence of the fourth exon of NM_006624 from the mouse transcript). PFAM [30] analysis of the proteins encoded by the two transcripts (NP_006615 and NP_653099) shows that the mouse protein lacks a N-terminus PHD finger domain that is present in the human protein. However, ASPicDB suggests a mouse transcript sharing exactly the structure of NM_006624 (602 aa) and encoding a protein with the complete PHD finger domain.

In Fig. 3B we show HTRA3 which has one annotated RefSeq transcript in human (NM_053044) and two in mouse (NM_030127 and NM_001042615). Mouse transcript NM_001042615 lacks two exons with respect to both NM_053044 and NM_030127, and as a consequence the protein it encodes (NP_001036080) lacks a C-terminus PDZ domain that has been characterized for the human HTRA3 protein (NP_444272) [31]. Nevertheless, ASPic predicts an isoform identical to NM_001042615 in human as well, thus suggesting that a HTRA3 protein lacking the PDZ domain is also produced in man.

Fig. 3C illustrates the potential of our proposed approach also for the identification of potentially erroneous transcript annotations. RAB15, a member of the RAS oncogene family, has one annotated RefSeq transcript both in human and mouse (NM_198686 and NM_134050, respectively). The fourth exon of the human transcript is longer than that in the murine mRNA and is responsible for a change of the reading frame of the subsequent exon with respect to the mouse transcript, leading to substantially different conceptual translation products. Analysis with PFAM indicates that the mouse protein contains the RAS domain typical of this family, while in the human protein this domain appears to be truncated. ASPic predicts a human isoform structurally identical to NM_134050 and encoding a protein with an intact RAS domain.

## Discussion

It is evident that the assignment of transcript orthology relationships based exclusively on reciprocal sequence similarity can produce misleading results if structurally different isoforms are inadvertently considered. To establish orthologous transcripts of orthologous genes in a reliable way, it is therefore important to take into account the conservation not only in sequence but also in intron-exon structure and in splicing pattern. This argument in itself raises the need to consider a new terminology for orthology of gene products, extending it to "orthologous splicing isoforms" for which we propose the name of "*iso-orthologous*" transcripts, that is, transcript pairs corresponding to the same splicing variant of orthologous genes. Here we have also presented evidence that current limited sampling of splicing isoforms complicates the comprehensive identification of orthologous splicing variants. However, the increase in the rate and economy of transcriptome sequencing with next-generation technologies is expected to rapidly fill this gap and to allow for more detailed studies of the evolution of alternative splicing, giving reliable estimates of the number of truly species- or taxon-specific alternative splicing events. In this respect, it should be considered that it has been observed that the number of detected AS isoforms steadily increase with the amount of available transcript data [14,15,32,33]. This suggests that a large fraction of AS events, particularly those which appear species-specific, are the result of background noise in the splicing process [34] and are likely non-functional. Consistently, it has been observed that the majority of lineage-specific exons are expressed at very low levels [14,15,35] even if, in some cases, the functional activity of minor isoforms has been assessed [24,36] and/or proteomic validation provided [37]. Therefore, extensive research activity is required to distinguish functional species-specific variants from non-functional splicing isoforms originating from neutral drift in the splicing process, taking into account that low-expressed non-functional isoforms provide the raw material for the evolutionary process and may acquire a function later.

The occurrence of potentially species-specific isoforms (and the implied evolution of patterns of alternative splicing) also raises at least another intriguing issue: whether novel splicing isoforms should be considered as deriving from existing splice patterns or directly from the gene of origin, in other words whether the evolution of alternative splicing can reasonably be depicted as a resolved tree-like structure.

## Conclusions

Our study clearly shows that it is important to extend the notion of orthology from genes to individual gene products. Multiple splicing variants derived from orthologous genes may or may not share the same exon/intron organization or encode for functionally equivalent products. We define here pairs of variants sharing the same pattern of splicing sites and exon/intron organization as "iso-structural orthologous splicing variants" or more briefly "iso-orthologs". These structurally similar variants are more likely to encode products with similar biological function, as in the case of p63 variants shown in Fig.1.

The large scale comparative analysis carried out in this study identified iso-orthologous pairs for most of human and mouse transcripts. However, for about 25% of human transcripts we were unable to identify a mouse iso-ortholog counterpart. The implications of this finding will be better clarified by the oncoming avalanche of transcriptome data from next-generation sequencing platforms. When a comprehensive transcriptome profile, in different cell types and conditions, will be available we will be able to better understand if missing iso-orthologous transcripts correspond to still unknown splicing variants or to species-specific transcript isoforms. Indeed, the identification of iso-orthologs should represent an important component of genome annotation strategies, comparative studies, phylogenetic analyses and functional genomics approaches, particularly given the fact that splicing variants can be lost or acquired in a taxon-specific manner during the evolutionary process.

## Methods

Gene structure was retrieved from the mapping of the RefSeq transcript collection available at the UCSC genome browser, March 2006 (NCBI36/hg18) for human and July 2007 (NCBI 37/mm9) for mouse. AspicDB transcript predictions were retrieved from version 2 (December 2009) [13]. Orthologous gene pairs were determined according to the annotations available in [27].

Alignments between intron/exon structures have been carried out using the Exalign algorithm [26]. The algorithm was run in global alignment mode, and allowing intron gain/loss detection so to exclude a wrong type assignation due to intron gain/loss events between the orthologous genes. The length of partially coding exons has been adjusted so to include only the coding portions. Fully non coding exons were excluded from the comparison.

The first round of analysis was performed by aligning the structure of every available RefSeq human transcript having at least two fully coding internal exons with all the available RefSeq mouse transcript structures from the corresponding orthologous gene. For each human RefSeq transcript only the highest scoring alignment has been considered for match type assignation. In an analogous way, each mouse RefSeq transcript structure has been aligned with all the RefSeq human transcript structures from the corresponding orthologous human gene.

Then, the same procedure was applied by adding AspicDB predictions to the set of RefSeq transcript structures. Whenever the highest scoring alignment for a given RefSeq transcript was derived from an alignment with an AspicDB prediction (and there was no RefSeq transcript producing an alignment with equal score), then the match type assignment was adjusted according to the one produced by the new alignment.

## Authors' contributions

FZA carried out the bioinformatics analyses, GPA participated in the design of the study, contributed to the bioinformatics analyses and in drafting the manuscript, CGI and DSH contributed to the conception of the study and critically revised the manuscript, GPE conceived the study and drafted the final version of the manuscript. All Authors read and approved the final manuscript.

## Supplementary Material

Additional file 1 **Full list of human-mouse and mouse-human iso-orthologous transcripts**. The additional file is an Excel spreadsheet consisting of two sheets: *sheet 1 *- orthologous splicing isoforms in the human - mouse comparison; *sheet 2 *- orthologous splicing isoforms in the mouse-human comparison. Click here for file
